# Silicon improves ion homeostasis and growth of liquorice under salt stress by reducing plant Na^+^ uptake

**DOI:** 10.1038/s41598-022-09061-8

**Published:** 2022-03-24

**Authors:** Zihui Shen, Xiaozhen Pu, Shaoming Wang, Xiuxiu Dong, Xiaojiao Cheng, Moxiang Cheng

**Affiliations:** 1 College of Life Sciences, Shihezi University, Shihezi, 832003 China; 2 Key Laboratory of Xinjiang Phytomedicine Resource and Utilization, Pharmacy School, Ministry of Education, Shihezi University, Shihezi, 832003 China

**Keywords:** Ecology, Plant sciences

## Abstract

Silicon (Si) effectively alleviates the effects of salt stress in plants and can enhance salt tolerance in liquorice. However, the mechanisms by which Si improved salt tolerance in liquorice and the effects of foliar application of Si on different liquorice species under salt stress are not fully understood. We investigated the effects of foliar application of Si on the growth, physiological and biochemical characteristics, and ion balance of two liquorice species, *Glycyrrhiza uralensis* and *G. inflata*. High salt stress resulted in the accumulation of a large amount of Na^+^, decreased photosynthetic pigment concentrations, perturbed ion homeostasis, and eventually inhibited both liquorice species growth. These effects were more pronounced in *G. uralensis*, as *G. inflata* is more salt tolerant than *G. uralensis*. Foliar application of Si effectively reduced the decomposition of photosynthetic pigments and improved gas exchange parameters, thereby promoting photosynthesis. It also effectively inhibited lipid peroxidation and leaf electrolyte leakage and enhanced osmotic adjustment of the plants. Furthermore, Si application increased the K^+^ concentration and reduced Na^+^ absorption, transport, and accumulation in the plants. The protective effects of Si were more pronounced in *G. uralensis* than in *G. inflata*. In conclusion, Si reduces Na^+^ absorption, improves ion balance, and alleviates the negative effects of salt stress in the two liquorice species studied, but the effect is species dependent. These findings may help to develop novel strategies for protecting liquorice plants against salt stress and provide a theoretical basis for the evaluation of salt tolerance and the scientific cultivation of liquorice.

## Introduction

Soil salinisation is the main factor for inhibition of agricultural development worldwide^1^. It has been estimated that 20% of the total cultivated land and 33% of the irrigated agricultural land is affected by high salinity^1^. Ion toxicity and osmotic stress are the two main threats of salinity stress, leading to the deficiency of Ca^2^^+^ and K^+^, other nutrient imbalances, finally inhibiting the growth and development of plants^2^. Ion toxicity is caused by the massive accumulation of Na^+^ in leaves in salt environment^3^, which will disrupt the water and ion balance in plants, damage the organelle structure, and inhibit plant growth, leading to plant death^3^. According to some studies, ion toxicity caused by Na^+^ is more likely to cause irreversible damage to plant than osmotic stress^3–6^. Furthermore, in plants, an antagonistic relationship exists between Na^+^ and K^+^, whereby high concentrations of Na^+^ directly inhibit the absorption of K^+^ by the plasma membrane^1,7^. The importance of K^+^ in plant growth is second only to that of nitrogen, as the activation of more than 60 plant enzymes depends on K^+^^8^. Therefore, maintaining a high K^+^/Na^+^ ratio under salt stress is essential for improving the salt tolerance of plants^1^.

Osmotic stress produces high levels of reactive oxygen species (ROS) under salt stress. Excessive ROS destroy macromolecules, lead to lipid peroxidation, and eventually inhibit plant growth^9^. To avoid the harmful effects of ROS and adjust this imbalance, plants can preserve homeostasis of intracellular environment through enzymatic and non-enzymatic antioxidant systems^10^. Enzymatic antioxidants mainly include superoxide dismutase (SOD) and catalase (CAT), while non-enzymatic antioxidants mainly include the accumulation of compatible penetrants such as proline, soluble sugar, and soluble protein^11^.

Silicon (Si), which is usually absorbed by higher plants in the form of H_4_SiO_4_^12^, is the second most abundant element in the Earth’s crust after oxygen^13^. While it is considered a non-essential element for the growth and development of higher plants^14^, many studies have shown that Si plays an important role in alleviating biotic and abiotic stresses in plants^15–19^. Foliar spray is the most effective Si application method^18,20^. Importantly, excessive application of Si does not cause environmental pollution^12^.

Si accumulates in the cell wall of plants in the form of SiO_2_, which not only enhances the mechanical function of the cell wall but also acts as a physical barrier to reduce water loss and improve the ability of plants to resist adverse environmental conditions^21,22^. Si also regulates polyamine metabolism, enhances H^+^-ATPase activity in the plasma membrane and tonoplast, promotes Na^+^ excretion from the cell, and reduces Na^+^ translocation and damage to plants^6,23^. Furthermore, Si improves the activity of antioxidant enzymes (such as SOD and CAT), reduces leaf electrolyte leakage (LEL)^24^, improves cell membrane stability, increases the water content of plant tissues, and improves photosynthetic function to promote plant growth^25^. Moreover, the recently proposed the ‘apoplastic obstruction hypothesis’ model^26^, which further explains the mechanism of Si-mediated plant resistance to biotic and abiotic stresses.

*Glycyrrhiza uralensis* Fisch. (*G. uralensis*) and *Glycyrrhiza inflata* Bat. (*G. inflata*) are the basic liquorice plants listed in the Chinese Pharmacopoeia^27^. Their roots and rhizomes have various pharmacological activities, such as anti-tumour^28^, anti-viral^29^, and anti-inflammatory^30^ effects. Furthermore, liquorice plants survive in extreme environments, such as those characterised by low temperature, drought, and high salinity and alkalinity. Accordingly, the soil of the liquorice habitat is usually mildly, moderately, or severely salinised. The total salt content in the soil suitable for *G. uralensis* growth is 0–0.7% and that suitable for *G. inflata* is 0–1.3%^31^. Consequently, liquorice is an important plant resource for improving saline–alkali soil and preventing wind and sand erosion in the arid and semi-arid regions of Northwest China^32^.

Xinjiang is a vast territory and the main producer of liquorice in China. However, around 31% of the existing cultivated land therein is damaged by salinity, so that 18% of the land is severely salinised and 33% shows medium-level salinisation, which is detrimental to the growth of liquorice^31^. According to recent studies^32,33^, at salt concentration exceeding 100 mmol L^–1^, the germination rate, germination potential, and germination index of *G. uralensis* seeds significantly decrease with a prolonged germination time. And the plant height, chlorophyll (Chl) content, and antioxidant enzyme activity of the seedlings are significantly reduced under this condition, eventually leading to low biomass and low effective component accumulation in *G. uralensis*^32–35^.

The ameliorating effect of Si application on plants under salt stress has been reported for many commercial crops, such as rice^17^, wheat^6^, sunflower^11^, sorghum^36^, and maize^5^, but the mechanisms involved vary among plant species. Further, to date, the understanding of the mechanism by which Si improves the salt tolerance of liquorice is limited, and it is not known whether the application of Si differently affects liquorice species. In the current study, we focused on *G. uralensis* and *G. inflata* in Xinjiang and explored the effects of foliar spray of Si on the growth, physiological and biochemical characteristics, and ion balance of liquorice under salt stress. We aimed to clarify the mechanisms whereby Si alleviates salt stress in these two liquorice species. This study provides a theoretical basis for the evaluation of salt tolerance and the scientific cultivation of liquorice.

## Materials and methods

### Plant material and growth conditions

A pot experiment was performed on the experimental field of the College of Life Science (Shihezi University, China; 44°30 N, 86°06 E). This region experiences a temperate continental climate, with a mean annual rainfall of 166 mm, a mean annual temperature of 25.6 °C, 169 frost-free days, and annual sunshine of 2769 h. *Glycyrrhiza uralensis* Fisch. seeds were collected from Wenquan County (Xinjiang, China; 44°58 N, 80°57 E), and *G. inflata* Bat. seeds were collected from Korla (Xinjiang, China; 41°69 N, 86°12 E). The composition of sandy soil was as follows: pH 7.63; silica, 30 mg kg^–1^; soluble salt, 2.9 g kg^–1^; total nitrogen, 0.246 g kg^–1^; total phosphorus, 0.105 g kg^–1^; total potassium, 5.988 g kg^–1^; available nitrogen, 42.549 mg kg^–1^; available phosphorus, 4.231 mg kg^–1^; available potassium, 82.509 mg kg^–1^; and soil organic matter, 5.826 g kg^–1^.

## Experimental design

The treatments were arranged in a completely randomised block design with three replicates. To explore the effects of leaf application of Si on the two liquorice species under different salt concentrations, based on the preliminary results, the following treatments were chosen for detailed analysis: (1) control (CK); (2) control + 3 mM Si (CK + Si); (3) 6 g kg^–1^ NaCl (6S); (4) 6 g kg^–1^ NaCl + 3 mM Si (6S + Si); (5) 12 g kg^–1^ NaCl (12S); and (6) 12 g kg^–1^ NaCl + 3 mM Si (12S + Si). Si was applied as a foliar spray in the form of K_2_SiO_3_ in Si treatments. To avoid the influence of osmotic pressure caused by K^+^ when K_2_SiO_3_ was added, 6 mM KCl was added to the control and treatment without Si, respectively. When the content of Cl^-^ was low, its influence on plant growth could be ignored^37^.

Ten seeds of liquorice were sown per plastic pot (23.5 × 16 × 18 cm^3^) containing 5 kg of sandy soil. After the emergence of 2 or 3 true leaves (15 d after sowing), 6 robust and disease-free liquorice seedlings of the same height and growth were retained in each pot and watered every day. NaCl and Si treatments were conducted simultaneously 4 weeks after sowing. Except for the treatments, other management measures were consistent with those of local field management practices. After 100 days of treatment, the growth parameters and physiological and biochemical characteristics of each liquorice species were determined.

### Determination of growth parameters

Plant height was measured using a tape measure, and stem thickness was measured using a Vernier calliper. The leaves, stems, and roots of liquorice were washed and placed in an oven at 105 °C for 30 min, dried at 75 °C for 48 h, and weighed.

### Determination of gas exchange attributes

Net photosynthesis rate (*P*_*n*_), transpiration rate (*T*_*r*_), stomatal conductance (*g*_*s*_), and intercellular CO_2_ concentration (*C*_*i*_) were recorded between 9:00 am and 12:00 am using a Li-6400 photosynthesis instrument (Li-COR, Lincoln, NE, USA). Three similarly sized healthy and fully expanded leaves from the top of the stem from plants under each treatment were analysed at a leaf temperature of 28 °C, irradiance of 1200 μmol m^–2^ s^–1^, and CO_2_ concentration of 400 μmol mol^–1^.

### Determination of photosynthetic pigments

The veins of fresh leaves were removed, cut into pieces, and weighed. Then, 10 mL of 80% (v/v) acetone was added to 0.5 g of the plant material and extracted in the dark until the leaves were colourless. Next, the supernatant was obtained after centrifugation at 4000 r min^−1^ for 10 min. Then we added 4 mL of 80% acetone to 1 mL of supernatant, and the absorbance values of the extracts were determined at 470 nm, 646 nm, and 663 nm using a Shimadzu UV-1900 spectrophotometer (Kyoto, Japan). The concentrations of Chl *a*, Chl *b*, total Chl, and carotenoids were calculated according to Lichtenthaler and Wellburn^38^.

### Determination of Na^+^ and K^+^ concentrations, transfer, and absorption

Dry plant samples (0.1 g; various portions, as specified) were digested in a mixture of nitric acid and perchloric acid (volume ratio 2:1). The leaf and root concentrations of Na^+^ and K^+^ were measured using a flame photometer (FP640, Shanghai Precision Scientific Instrument Co., Ltd., Shanghai, China). Sodium uptake at the liquorice root surface and ion (Na^+^ and K^+^) translocation from the root to shoot were calculated using the methods described by Yan et al*.*^39^ and Ali et al*.*^5^, respectively.

### Determination of soluble sugar, soluble protein, and proline contents

For soluble sugar determination, fresh leaves (0.2 g) were ground into a homogenate in 6 mL of distilled water and then incubated in a water bath at 100 °C for 20 min. After cooling, the samples were centrifuged at 3000 r min^–1^ for 10 min. The extract (1.0 mL) was then mixed with 5 mL of anthrone reagent, and the absorbance value at 620 nm was measured using a Shimadzu UV-1900 spectrophotometer (Kyoto, Japan). The soluble sugar content was then calculated using a standard curve of sucrose^40^.

Soluble protein was determined using the Coomassie Brilliant Blue G-250 method^41^. Fresh leaves (0.2 g) were added to phosphate buffer solution (pH 7.0), ground into a homogenate, and centrifuged at 5000 r min^–1^ for 10 min. Coomassie Brilliant Blue G-250 reagent was added to 1 mL of the supernatant and the absorbance was read at 595 nm. The protein content was calculated using a standard curve of bovine serum albumin^40^.

For proline content determinations, fresh liquorice leaves (0.5 g) were placed in 5 mL of 3% sulfosalicylic acid solution and centrifuged at 5000 r min^–1^ for 10 min. The supernatant (2 mL) was added to 2 mL of glacial acetic acid and 2 mL of acidic ninhydrin reagent, and the mixture was heated in a boiling water bath for 30 min. After cooling, 4 mL of toluene was added, shaken for 30 s, and centrifuged at 5000 r min^–1^ for 10 min. Using toluene as a blank control, sample absorbance was measured at 520 nm by using a Shimadzu UV-1900 spectrophotometer (Kyoto, Japan), and the proline content was calculated using a standard curve by Li^40^.

### Determination of lipid peroxidation and LEL

Lipid peroxidation was determined by measuring the concentration of malondialdehyde (MDA). Liquorice leaves (0.5 g) were homogenised in 5 mL of 0.1% (w/v) trichloroacetic acid solution. After centrifugation at 10,000 r min^–1^ for 10 min, the supernatant was mixed with 0.5% (w/v) trichloroacetic acid solution and incubated in a water bath at 100 °C for 2 min. The samples were then centrifuged at 10,000 r min^–1^ for 10 min. The absorbance of the sample supernatant at 600 nm, 532 nm, and 450 nm was determined using a Shimadzu UV-1900 spectrophotometer (Kyoto, Japan), and the MDA content was calculated as described by Li^40^.

To determine the LEL, fresh liquorice leaves were cleaned, cut into 2 cm pieces, and placed in a test tube containing 10 mL of distilled water. The samples were shaken on an oscillating table at 25 °C for 24 h to determine electrical conductivity (EC_1_). The test tube was then placed in a water bath at 100 °C for 30 min to determine electrical conductivity (EC_2_)^42^. LEL was calculated using the following formula: LEL (%) = EC_1_/EC_2_ × 100%.

### Determination of antioxidant enzyme activities

Fresh leaves (0.5 g) were added to phosphate buffer solution (pH 7.0), ground into a homogenate, and centrifuged at 10,000 r min^–1^ for 10 min. The supernatant was diluted to 25 mL using the same buffer solution. The samples were then stored in an icebox for determination of SOD and CAT activities. All operations were performed at 0–4 °C. SOD activity was determined using the nitrogen blue tetrazole method^40^, and CAT activity was determined using the colorimetric method^43^.

### Statistical analysis

Analysis of variance was used to test the effect of different treatments on each index in the same liquorice species (P < 0.05), and Duncan’s multiple comparisons test was used to determine significant differences between different treatments of the same liquorice. Meanwhile, data were checked for normality and the homogeneity of variances and the data of SOD and intercellular CO_2_ concentration were transformed with natural logarithm to correct deviations from these assumptions, when needed. The overall data were analysed using SPSS 20.0 (SPSS Inc., Chicago, IL, USA).

### Compliance statement for experimental materials

Liquorice is a widely distributed species in China. Xinjiang is the main producing area of liquorice in China. Seed of *G. uralensis* was collected from Wenquan County (Xinjiang, China; 80°57E, 44°58 N), and that of *G. inflata* was collected from Korla (Xinjiang, China; 86°12E, 41°69 N). A pot experiment was performed on the experimental field of the College of Life Science (Shihezi University, China; 86°06E, 44°30 N). Therefore, all operations comply with relevant institutional, national, and international guidelines and legislation.

## Results

### Plant growth

Salt stress significantly inhibited the growth of the two liquorice species. The inhibitory effect on plant growth was more pronounced in *G. uralensis* than in *G. inflate*. Under the 12S treatment, where 12 g kg^–1^ NaCl was used, both species displayed significant reductions in plant height, root dry weight, and shoot dry weight compared to their respective controls. However, between the two species, *G. inflata* showed better plant height, root dry weight, and shoot dry weight by 57%, 39%, and 42%, respectively, than *G. uralensis* (Table 1). Nevertheless, this marked inhibitory effect in *G. uralensis* was alleviated by Si (Table 1). Under the 12S + Si treatment, plant height, root dry weight, and shoot dry weight of *G. uralensis* increased by 74%, 81%, and 74%, respectively, compared to the corresponding values without Si. However, in the case of Si treatment of *G. inflata*, these indicators increased by 19%, 48%, and 49%, respectively compared to those of the control. The foliar application of Si resulted in a significant increase in growth characteristics of both the species. The growth enhancement was markedly higher in *G. uralensis* than *G. inflata*.

**Table 1:** Effects of silicon on the growth of *Glycyrrhiza uralensis* and *G. inflata* under salt stress.

Cultivar	Treatment	Plant height (cm)	Stem diameter (mm)	Root DW (g)	Shoot DW (g)
*G. uralensis*	CK	25.53 ± 0.46b	2.06 ± 0.11b	3.09 ± 0.10b	2.34 ± 0.06a
	6S	18.6 ± 0.72c	1.75 ± 0.06bc	1.77 ± 0.04d	1.67 ± 0.03b
	12S	11.43 ± 0.07d	1.48 ± 0.07c	0.36 ± 0.06e	0.38 ± 0.07d
	CK + Si	33.20 ± 0.85a	2.95 ± 0.18a	3.85 ± 0.21a	2.51 ± 0.11a
	6S + Si	26.30 ± 0.06b	2.15 ± 0.12b	2.16 ± 0.14c	1.74 ± 0.06b
	12S + Si	19.87 ± 0.34c	1.89 ± 0.16b	0.66 ± 0.04e	0.66 ± 0.05c
*G. inflata*	CK	30.47 ± 0.78b	2.31 ± 0.04c	3.54 ± 0.04b	3.20 ± 0.09a
	6S	22.33 ± 0.94d	1.97 ± 0.02d	2.80 ± 0.05c	2.26 ± 0.13b
	12S	18.00 ± 1.26e	1.67 ± 0.02e	0.51 ± 0.06e	0.53 ± 0.07c
	CK + Si	38.97 ± 0.74a	2.87 ± 0.06a	4.24 ± 0.07a	3.42 ± 0.21a
	6S + Si	26.83 ± 1.19c	2.45 ± 0.05b	3.40 ± 0.02b	2.34 ± 0.22b
	12S + Si	21.33 ± 0.93d	1.84 ± 0.05 cd	0.75 ± 0.04d	0.80 ± 0.10c

Data are presented as the mean ± SE (n = 3). Different letters indicate significant differences between the values for the same index for the same liquorice species under different treatments (P < 0.05). DW, dry weight.

### Gas exchange attributes

**Figure 1:**
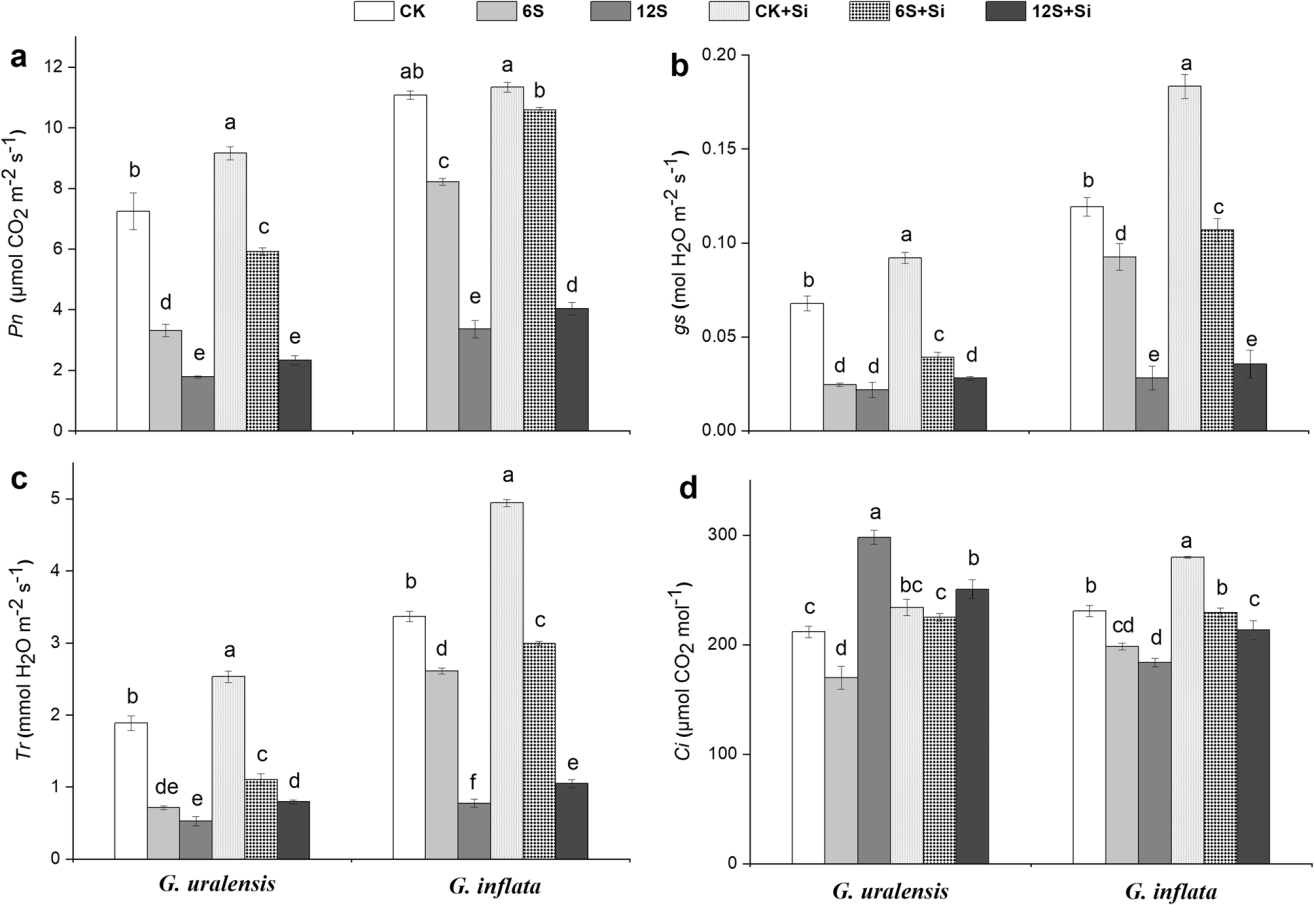
Effects of salinity and Si on net photosynthetic rate (*Pn*, **a**), stomatal conductance (*gs*, **b**), transpiration rate (*Tr*, **c**), and intercellular CO_2_ concentration (*Ci*, **d**) in the two liquorice species. Data are presented as the mean ± SE (n = 3). Different letters indicate significant differences between the values for one index for the same liquorice species under different treatments (P < 0.05).

Salt treatment (6S and 12S) showed a reduction in gas exchange attributes with increasing NaCl concentrations (Fig. 1). However, in the case of 12S salt treatment, G. *uralensis* showed a 41% increase in *C*_*i*_ compared to that in the untreated control (Fig. 1d). The various gas exchange parameters analysed indicated an overall enhancement with respect to Si treatment in both the species, irrespective of the NaCl concentration. A similar result was observed for the CK + Si plants that showed a marked increase in all the gas exchange parameters with Si treatment. However, in the 12S + Si treatment, *G. uralensis* showed a 16% reduction in the *C*_*i*_ levels (Fig. 1d). Between the two species, *G. uralensis* showed greater increase in gas exchange parameters in the 6S + Si treatment. *G. uralensis* showed a 78% increase (Fig. 1a) in *P*_*n*_ compared to that of *G. inflata*, which only showed a 29% increase in *P*_*n*_. In *G. uralensis*, *g*_*s*_ increased by 59% (Fig. 1b) compared to a 15% increase in *G. inflata*. Furthermore, *G. uralensis* showed a 55% increase (Fig. 1c) in *T*_*r*_ and a 33% increase in *C*_*i*_ (Fig. 1d), whereas *G. inflata* showed only a 15% increase for both the parameters.

### Photosynthetic pigments concentrations

**Figure 2:**
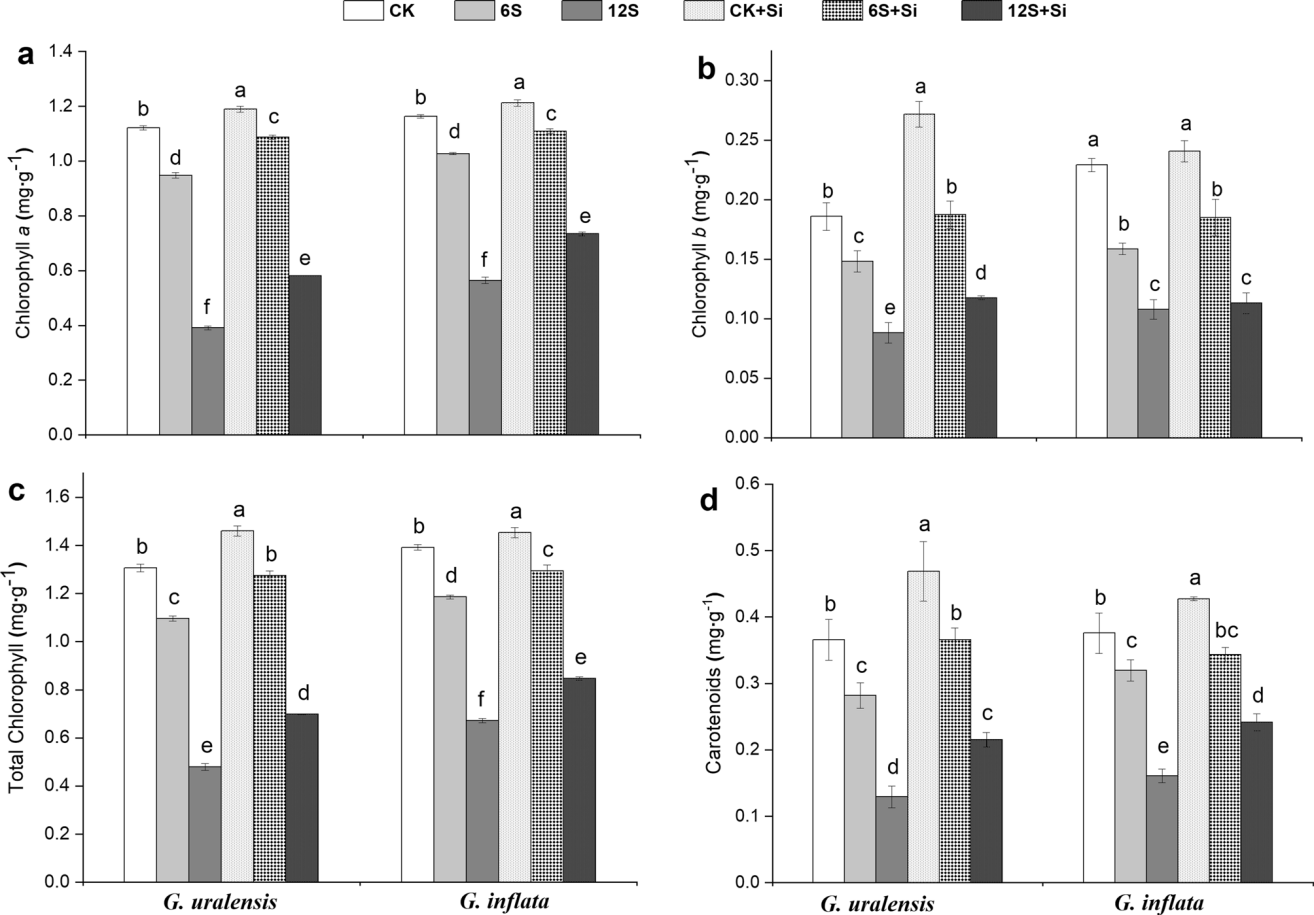
Effects of salinity and Si on chlorophyll *a* **(a)**, chlorophyll *b* **(b)**, total chlorophyll **(c)**, and carotenoids content **(d)** in the two liquorice species. Data are presented as the mean ± SE (n = 3). Different letters indicate significant differences between the values for one index for the same liquorice species under different treatments (P < 0.05).

Salt treatment significantly reduced the photosynthetic pigments concentrations in both the liquorice species compared to those in CK (Fig. 2). However, Si treatment (CK + Si, 6S + Si, and 12S + Si) resulted in a marked increase in chlorophylls and carotenoids concentrations in both control and salt-treated plants (Fig. 2a–d). In the 12S + Si treatment, *G. inflata* showed a marginal 5% increase of Chl *b* when compared to *G. uralensis*, which showed a 33% increase of Chl *b* (Fig. 2b).

### Accumulation of Na^+^ and K^+^ in the root and leaf

**Figure 3:**
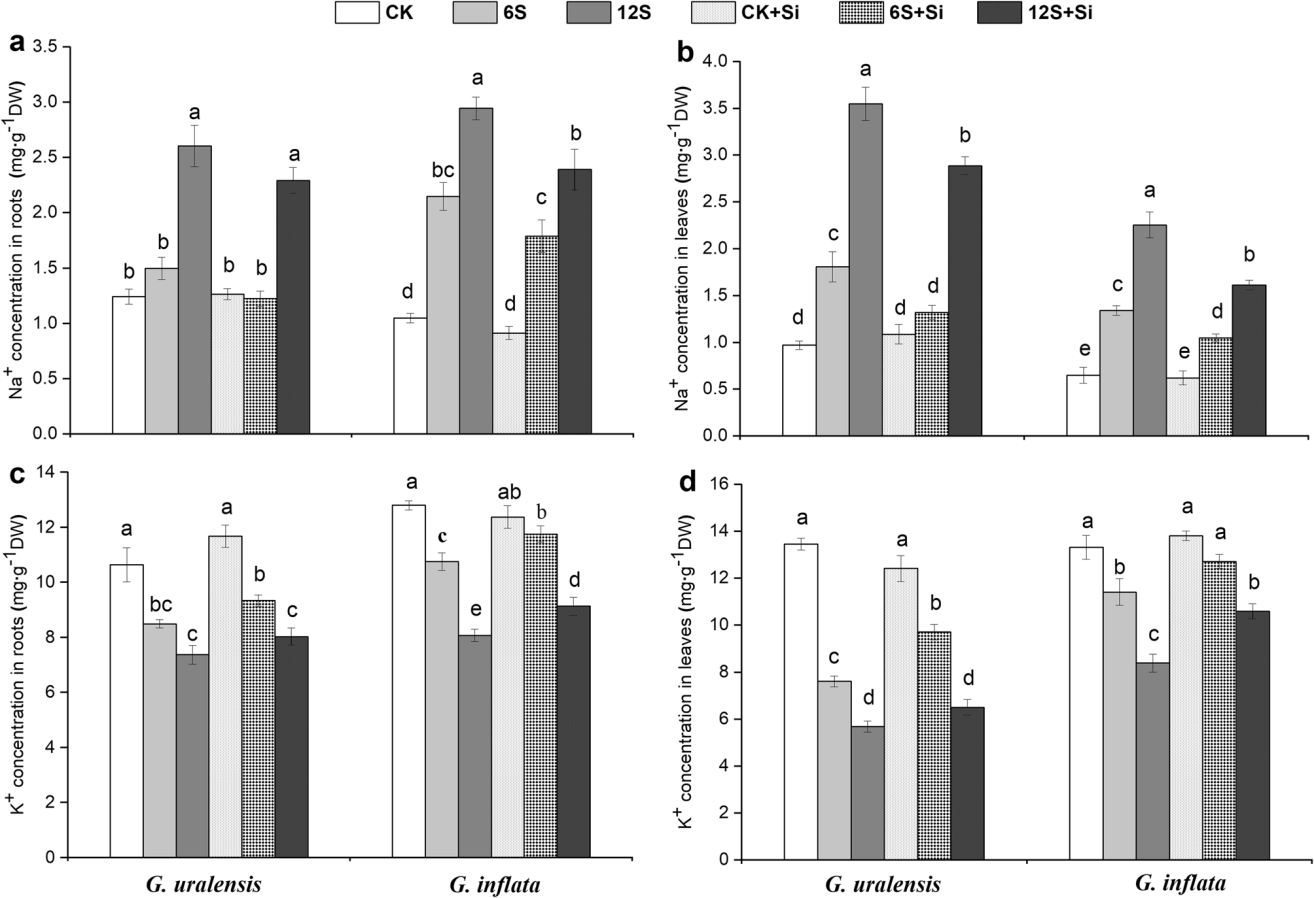
Effects of salinity and Si on Na^+^ concentration in the root **(a)** and leaf **(b)**, and K^+^ concentration in the root **(c)** and leaf **(d)** in the two liquorice species. Data are presented as the mean ± SE (n = 3). Different letters indicate significant differences between the values for one index for the same liquorice species under different treatments (P < 0.05).

The accumulation of Na^+^ in the root and leaf of the two liquorice species increased significantly with increasing salt concentrations, with a simultaneous significant decrease in the accumulation of K^+^ (Fig. 3). *G. uralensis* showed a significant increase in the accumulation of Na^+^ in the leaf tissue compared to that of *G. inflata*. The CK, 6S, and 12S salt treatments resulted in a 49%, 35%, and 57% increase in Na^+^ accumulation, respectively, in *G. uralensis* (Fig. 3b). Si application caused a significant reduction in the accumulation of Na^+^ in the root and leaf tissues of both the species in the 6S as well as 12S salt treatments (Fig. 3a,b). The application of Si with the 6S treatment resulted in a 27% reduction in Na^+^ accumulation in the leaves of *G. uralensis* (Fig. 3b) and a subsequent 28% increase in K^+^ accumulation (Fig. 3d).

### Ion translocation and uptake

**Figure 4:**
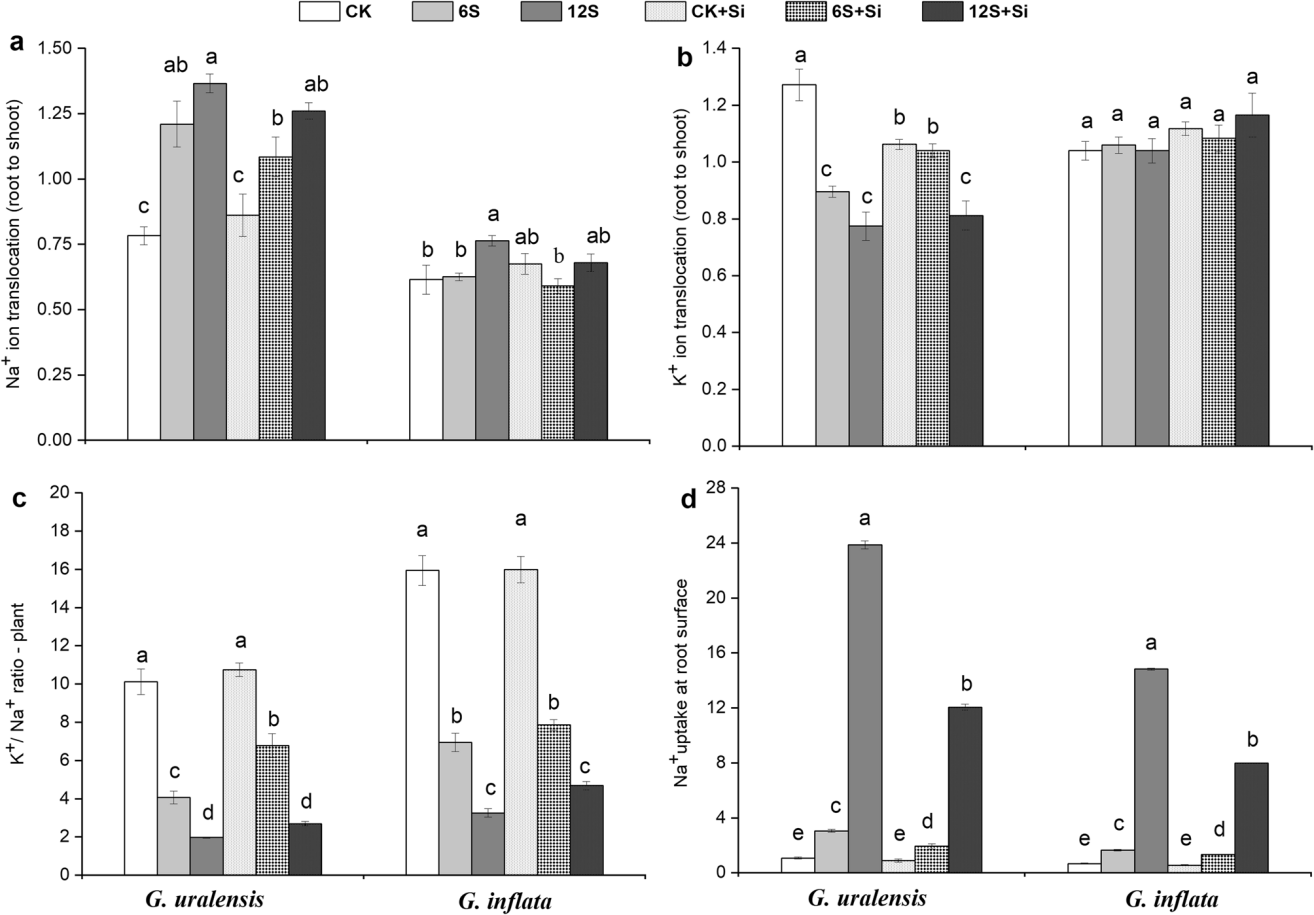
Effects of salinity and Si on Na^+^ translocation **(a)**, K^+^ translocation **(b)**, K^+^/Na^+^ ratio **(c)**, and Na^+^ uptake at root surface **(d)** in the two liquorice species. Data are presented as the mean ± SE (n = 3). Different letters indicate significant differences between the values for one index for the same liquorice species under different treatments (P < 0.05).

Salt treatment significantly increased Na^+^ transport (root–leaf) and Na^+^ uptake on the root surface and decreased the K^+^/Na^+^ ratio in both the liquorice species (Fig. 4). K^+^ transport and K^+^/Na^+^ ratio in *G. inflata* were 50% and 71% higher than those in *G. uralensis* under the 6S treatment, respectively, while Na^+^ transport and Na^+^ uptake on the root surface of *G. uralensis* were 35% and 93% higher than those of *G. inflata*, respectively. The application of Si decreased the Na^+^ transport and increased the K^+^ transport and K^+^/Na^+^ ratio, thereby significantly affecting the uptake of Na^+^ on the root surface under 12S treatment. 12S + Si treatment reduced the uptake of Na^+^ by 49% in *G. uralensis* and 46% in *G. inflata*.

### Soluble sugar, soluble protein, and proline contents

**Figure 5:**
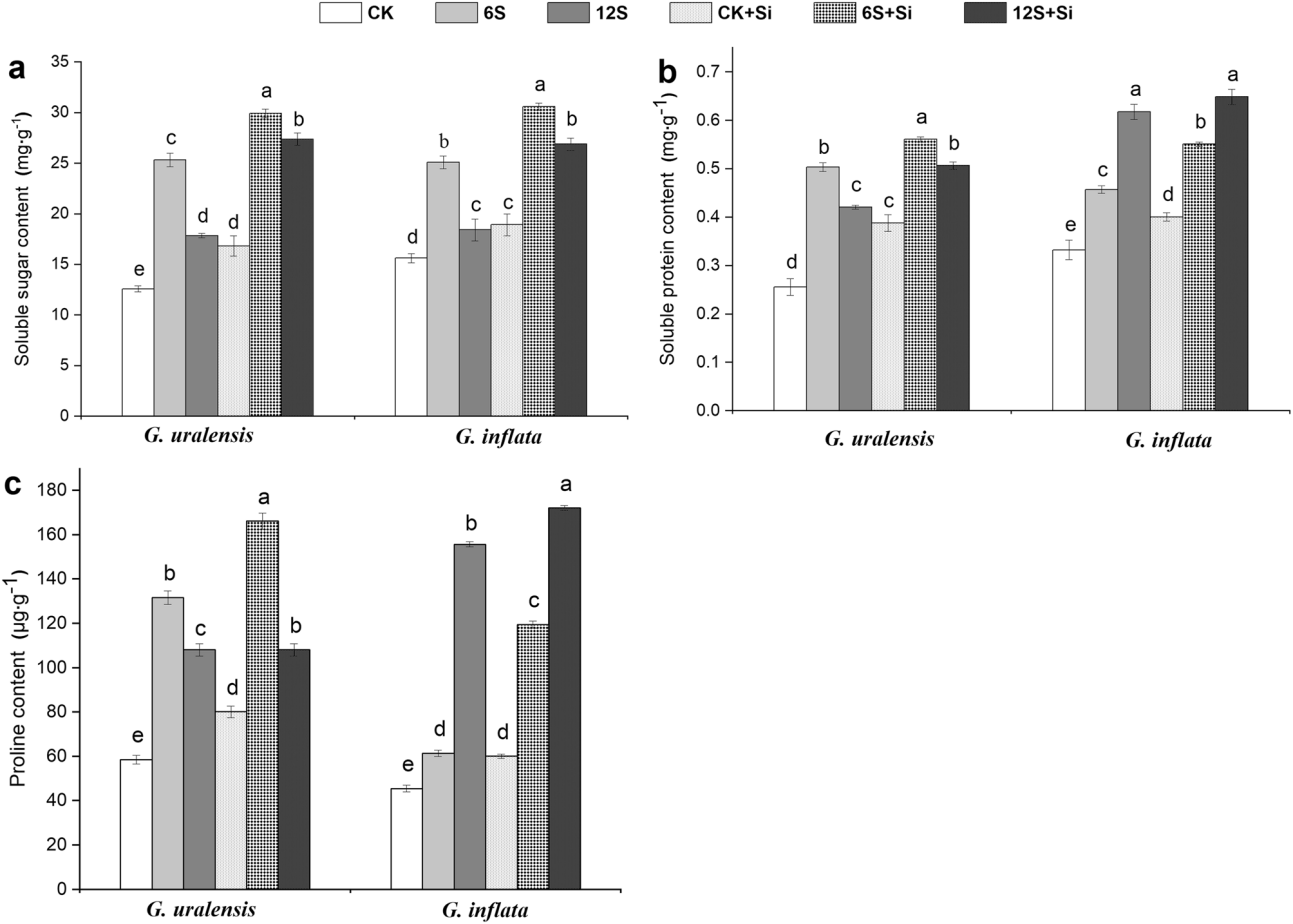
Effects of salinity and Si on soluble sugar content **(a)**, soluble protein content **(b)**, and proline content **(c)** in the two liquorice species. Data are presented as the mean ± SE (n = 3). Different letters indicate significant differences between the values for one index for the same liquorice species under different treatments (P < 0.05).

Salt treatment resulted in a significant increase in the accumulation of soluble sugars in both the species (Fig. 5a) with the maximum accumulation in the 6S samples. In the 6S samples, soluble sugars of *G. uralensis* and *G. inflata* were 101% and 61% higher than those in the CK samples, respectively. Si application also resulted in 18% and 22% increase of sugars accumulation in the 6S + Si samples of both the two species. The accumulation of soluble protein (Fig. 5b) was the maximum in the 6S samples with a 97% increase in *G. uralensis* and a 38% increase in *G. inflata*. Si application resulted in an increase in soluble protein concentration in both the species. Compare with the CK samples, accumulation of proline (Fig. 5c) was the highest in the 6S samples for *G. uralensis* by 125%, whereas for *G. inflata*, the maximum accumulation of proline was obtained in the 12S samples by 242%. In addition, Si application resulted in an increase in proline concentrations in both the species across all salt treatments. Especially at the 6S + Si treatment, where the accumulation of proline increased by 26% for *G. uralensis* and by 94% for *G. inflata*, as compared with 6S treatment alone.

### Lipid peroxidation and LEL

**Figure 6:**
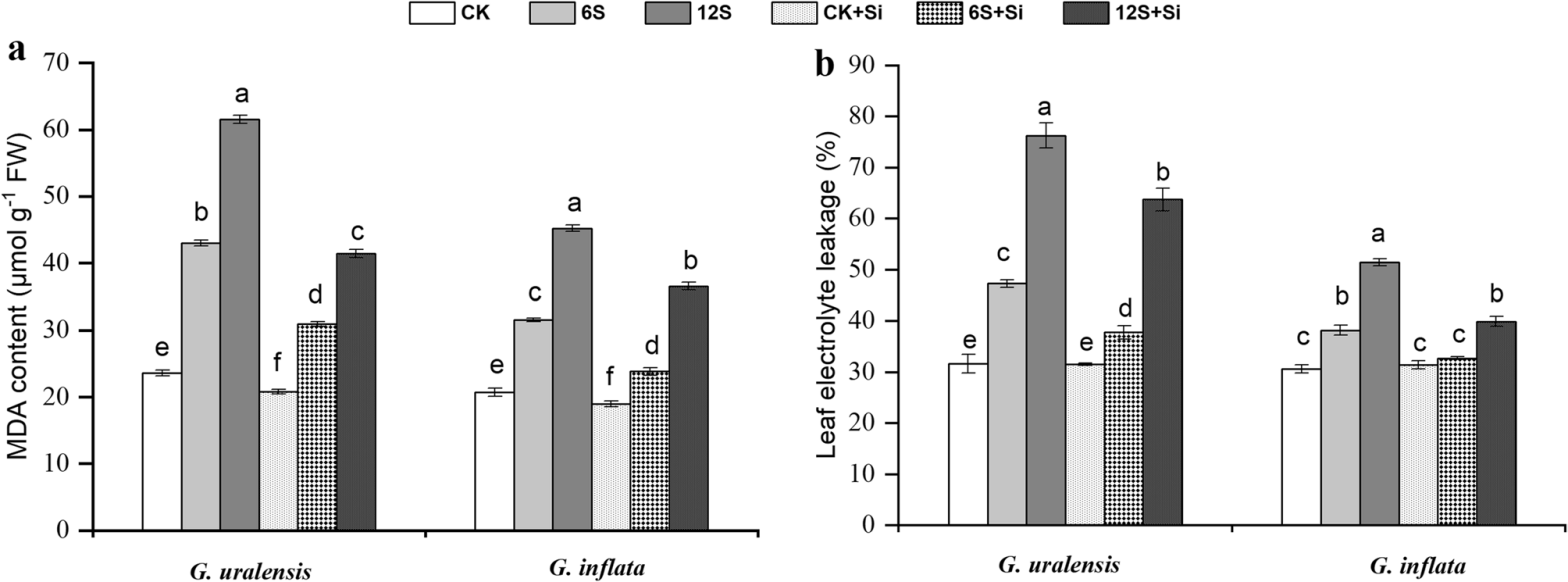
Effects of salinity and Si on malondialdehyde content (MDA, **a**) and leaf electrolyte leakage (LEL, **b**) in the two liquorice species. Data are presented as the mean ± SE (n = 3). Different letters indicate significant differences between the values for one index for the same liquorice species under different treatments (P < 0.05).

The accumulation of MDA and LEL significantly increased with increase in salt concentrations in both the liquorice species. The changes were more pronounced in *G. uralensis* than in *G. inflata* (Fig. 6). Si application resulted in a significant reduction in the MDA levels and LEL in both the species. In the 12S + Si treatment, Si application reduced the accumulation of MDA by 33% in *G. uralensis* and 19% in *G. inflata*. Similarly, Si application significantly reduced LEL by 16% in *G. uralensis* and 22% in *G. inflata*.

### Antioxidant enzyme activities

**Figure 7:**
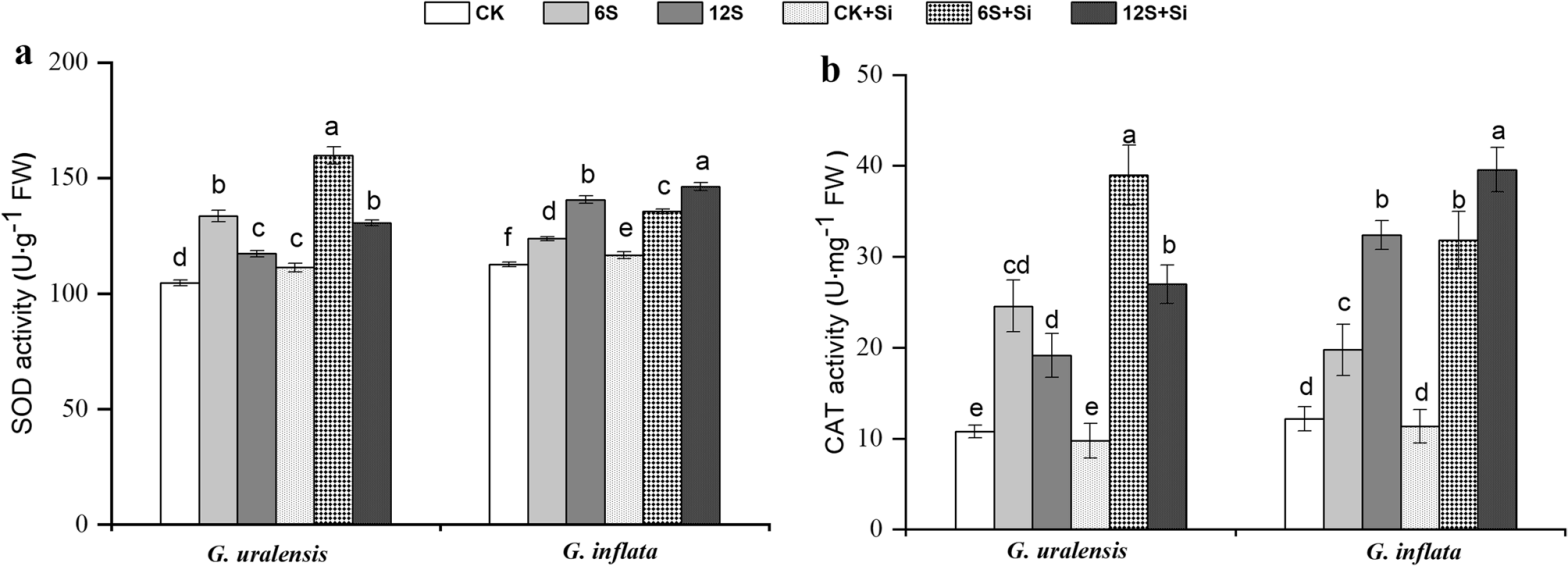
Effects of salinity and Si on superoxide dismutase activity (SOD, **a**) and catalase activity (CAT, **b**) in the two liquorice species. Data are presented as the mean ± SE (n = 3). Different letters indicate significant differences between the values for one index for the same liquorice species under different treatments (P < 0.05).

SOD and CAT activities of the two species were both increased at 6S and 12S treatments compared with the CK, which for *G. uralensis* showed the maximum activity of 28% and 128% in the 6S treatment, whereas those for *G. inflata* were 25% and 166% in the 12S treatment (Fig. 7). However, Si application resulted in increased SOD and CAT activities across all salt treatments in both the species. The exogenous application of Si under 6S treatment improved the activities of SOD and CAT in *G. uralensis* by 20% and 59%, while the increment was about 10% and 61% respectively in *G. inflata*, as compared with 6S treatment alone.

### Correlation analysis

**Figure 8:**
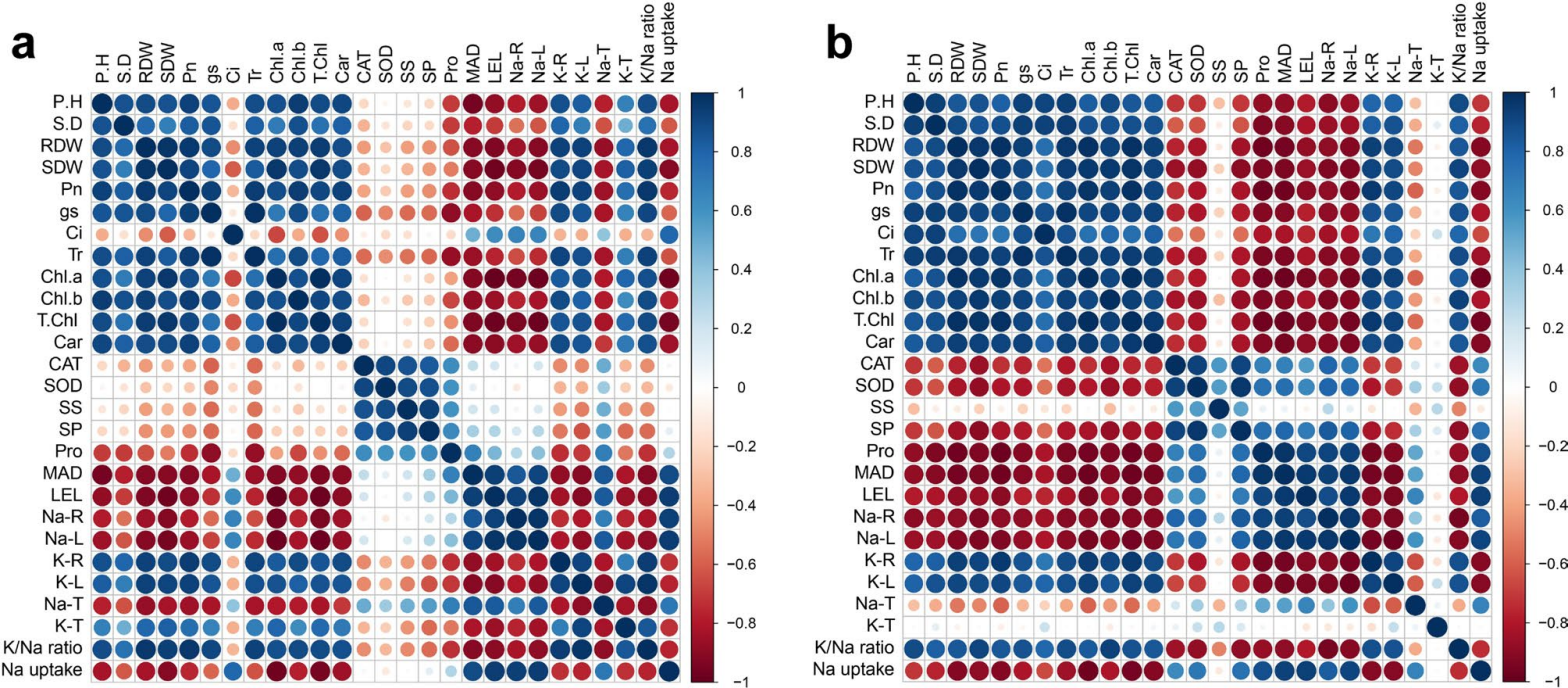
Correlation analysis (P < 0.05) between various measured attributes of *Glycyrrhiza uralensis* **(a)** and *G. inflata* **(b)**. The abbreviations are as follows: PH (plant height), SD (stem diameter), SDW (shoot dry weight), RDW (root dry weight), *Pn* (net photosynthesis rate), *Tr* (transpiration rate), *Gs* (stomatal conductance), *Ci* (internal CO_2_ concentration), Chl *a* (chlorophyll *a*), Chl *b* (chlorophyll *b*), T. Chl (total chlorophyll), Car (carotenoid content), SOD (superoxidase activity), CAT (catalase activity), SS (soluble sugar content), SP (soluble protein content), Pro (proline content), MDA (malondialdehyde content), LEL (leaf electrolyte leakage), Na-R (sodium concentration in roots), Na-L (sodium concentration in leaves), K-L (potassium concentration in leaves), K-R (potassium concentration in roots), Na-T (Na^+^ translocation), K-T (K^+^ translocation), K/Na ratio (K^+^/Na^+^ ratio), Na uptake (Na^+^ uptake).

Pearson’s correlation analysis was performed to monitor the differences in plant growth, physiology, and biochemical attributes along with ion homeostasis of *G. uralensis* and *G. inflata* (Fig. 8). The concentration of Na^+^ in liquorice roots showed a positive correlation with Na^+^ in the leaves, Na^+^ absorption on the root surface, and oxidative stress indexes MDA and LEL. Na^+^ in liquorice roots showed a negative correlation with growth indexes, gaseous exchange attributes (except *Ci* for *G. uralensis*), Chl content, K^+^ in the roots and leaves, and K^+^/Na^+^ ratio.

## Discussion

Salinity stress is one of the main adverse environmental conditions encountered by plants^1^. The detrimental effects of salinity include ion toxicity and osmotic stress, which cause growth inhibition, yield reduction, and may eventually lead to plant death^44^. Several studies indicate^45,46^ that Si can be used to effectively alleviate the harmful effects of salt stress on plants and to promote plant growth in saline environments.

In the current study, we analysed the growth indicators, gas exchange parameters, Chl content, ion balance, osmotic regulators, membrane damage indicators, and antioxidant enzyme activity in the liquorice species *G. uralensis* and *G. inflata.* Although several studies have investigated the effect of Si on the growth of liquorice under salt stress^33–35,37,47^, this study demonstrates that exogenous application of Si by foliar spray enhances salt tolerance of *G. uralensis* and *G. inflata*.

Both medium-salt (6S) and high-salt (12S) treatments significantly reduced the biomass of the two liquorice species, with the lowest values obtained at high salt concentrations. Under the same salt treatment, the growth of *G. inflata* was better than that of *G. uralensis*, indicating that *G. inflata* is more salt tolerant than *G. uralensis*. However, the effects are evidently mitigated by foliar application of Si which resulted in increased biomass in both the species (Table 1).

We studied the gas exchange attributes of *G. uralensis* and *G. inflata* by analysing the *P*_*n*_, *T*_*r*_, *g*_*s*_, and *C*_*i*_ in both the liquorice species. Foliar application of Si alleviated the negative effects of salt stress on all these gas exchange parameters in the two liquorice species (Fig. 1). Under high salt stress, we observed a significant increase in the *C*_*i*_ levels in *G. uralensis* (Fig. 1d). This was an unexpected result; however, it is possible that the stomatal protection system is damaged when the salt concentration exceeds the tolerance limit of the species and can be attributed to the involvement of non-stomatal factors that play a major role in photosynthesis^48^. In addition, under high salt stress, the *P*_*n*_, *T*_*r*_, and *g*_*s*_ of the two liquorice species were also significantly reduced. However, all of these negative effects were ameliorated by the foliar application of Si in both the species, further establishing the role of Si in ameliorating the effects of salt treatment.

Consistent with the findings for mung bean^9^ and cucumber^19^, salt stress reduced photosynthetic pigments concentrations in the leaves of the two liquorice species (Fig. 2). However, foliar application of Si significantly increased chlorophylls and carotenoids concentrations in plant leaves under salt stress. These observations suggest that Si promotes the biosynthesis of photosynthetic pigments under salt stress. This may be attributed to the notion that Si alleviates the damage to chloroplast under salt stress^34^, enhances Rubisco protein expression, and promotes the synthesis of photosynthetic pigments^45^.

The negative effects of salt stress such as damage to plant cells and the decrease in photosynthesis rate are mainly caused by excessive absorption and accumulation of Na^+^^6,18^. When subjected to high salt stress, it is possible that the ability of the root system of *G. uralensis* to intercept Na^+^ is compromised, which led to the high accumulation of Na^+^ in the root and leaf tissues (Fig. 3). This in turn led to the disturbance in the synthesis of photosynthetic pigments^34^, a decrease in the net photosynthetic rate, and a significant reduction of plant biomass (Figs. 1a, 2, and Table 1). In high-salt environments, the accumulation of Na^+^ increases in the roots, and the transport of Na^+^ is enhanced towards the shoot, thus, increasing Na^+^ accumulation in the leaves, which damages the mesophyll cells^49^. When the Na^+^ concentration in the leaf exceeds 1.3 mg g^–1^, the chloroplast structure is damaged, Chl degradation is accelerated, and photosynthesis is inhibited^50^. Our results indicate that the transport and absorption of Na^+^ on the root surface were higher in *G. uralensis* than in *G. inflata* under medium-salinity conditions. This indicates that the ability of *G. inflata* roots to intercept Na^+^ is greater than that of *G. uralensis*, which might account for the relatively high salt tolerance of *G. inflata*.

K^+^ is the key regulator of cell homeostasis^8^ and plays an important role in inducing cell elongation, maintaining osmotic regulation in plants, and promoting photosynthesis^8,51,52^. Therefore, excessive Na^+^ levels often lead to K^+^ deficiency. Al-Huqail et al*.*^45^ reported that high salt concentrations significantly increase the Na^+^ content in maize and greatly reduce the K^+^ content, resulting in an increased Na^+^/K^+^ ratio and plant growth inhibition. In *G. uralensis*, compared with the application of Si in the soil, foliar spray alone effectively increased K^+^ levels and reduced Na^+^ levels^35^. This may be associated with the Si-induced upregulation of genes involved in potassium uptake (*OsAKT1* and *OsSHAK1*) and xylem load (*OsSKOR*)^53^, which promotes the increase of K^+^ transport and increases the H^+^-ATPase activity, forming a mechanical barrier to reduce Na^+^ transport^54^. Therefore, we believe that the capacity of Si to enhance K^+^-selective transport and increase the K^+^/Na^+^ ratio might be the main mechanisms to improve plant growth and productivity under salt stress, which is in agreement with the findings of previous studies^5,52^. In addition, Si not only reduced the Na^+^ transfer and damage to the shoot under salt stress but also decreased the Na^+^ absorption by the root in the two liquorice species (Fig. 3), which is consistent with previous findings in rice^17^ and wheat^6^. There are at least two possible explanations: (1) Si deposition on the root cell wall enhances the mechanical strength of the cell wall, thus, reducing Na^+^ absorption^13^; and (2) Si decreases Na^+^ accumulation in the root apex and cortex by upregulating the expression of *ZmSOS1* and *ZmSOS2* transporters^55^. Considering both scenarios, we could say that the reduction of Na^+^ uptake and transport may well be a potential mechanism of Si-mediated enhancement of salt tolerance in liquorice.

In high-salt environments, plants accumulate high levels of Na^+^. This leads to excessive accumulation of ROS, perturbing the balance between their production and elimination, and resulting in cellular oxidative damage^9^. MDA and LEL are the main indexes used to evaluate the severity of oxidative cell damage^17^. MDA is an oxidation product of membrane lipids and accumulates when plants are subjected to oxidative stress. Na^+^ accumulation results in the production of high levels of ROS, which destroy the cell membrane structure, leading to an increased MDA content and LEL. For instance, salt-induced oxidative damage leads to the rupturing of the plasma membrane in maize, leading to lipid peroxidation^5^. In this study, we showed that both medium and high-salt treatments increased the MDA content and LEL in the two liquorice species, especially in *G. uralensis* (Fig. 6), indicating major damage of the cell membrane in these liquorice species. Of the two species, the damage was more pronounced in *G. uralensis*. However, the MDA content and LEL in the two liquorice species decreased after foliar spraying with Si. These observations suggest that Si may counteract the membrane damage caused by salt stress in liquorice. In addition, Si can alleviate reactive oxygen damage by maintaining membrane integrity and activating the antioxidant defence system. In this study, Si enhanced SOD and CAT activities in the two liquorice species under salt stress (Fig. 7), which is consistent with the observations in mung bean^9^ and mustard^56^. This effect may be related to the upregulation of genes encoding antioxidant enzymes^57^. We also observed that Si application enhanced the SOD and CAT activities to a greater extent in *G. uralensis* than in *G. inflata*. Based on the observations in this study, we could say that the regulation of the plant antioxidant system by Si upon salt stress is different for the two different liquorice species.

The alleviating effect of Si described above was not only associated with the increased antioxidant enzyme activity but also with the accumulation of osmotic regulators in plants^45^. Accordingly, upon salt stress, plants produce and accumulate compatible organic solutes as a means of osmotic adjustment, to maintain the normal physiological and biochemical characteristics of intracellular water and to impede the damage to the cell membrane^48,58^. We demonstrated in this study that under high-salt stress, the soluble sugar, soluble protein, and proline contents in the leaf of *G. uralensis* were increased (Fig. 5), indicating that high-salt stress greatly affected the ability of *G. uralensis* to respond to the adverse effects of high-salt. Proline is a key osmoprotectant and can reduce the damage caused by ROS, reduce lipid peroxidation, and protect protein and membrane structures^48^. In the current study, the application of Si reduced the proline content in liquorice, which was consistent with the findings in sunflower^11^ and pelargonium^24^. In the presence of Si, proline was degraded and used as a source of carbon and nitrogen in plants recovering from stress, as well as a membrane stabiliser and a free-radical scavenger to reduce lipid peroxidation and LEL^16,20^. Therefore, reduction of membrane damage may be another mechanism whereby Si improves the salt tolerance of plants. However, the mechanism for obtaining Si to improve plant salt tolerance is a complex process. In addition to many physiological and biochemical aspects, further research is required on protein and gene expression analysis.

## Conclusions

High salinity greatly inhibited the growth and development of the two liquorice species studied. Under salt stress, the morphological characteristics (plant height, stem diameter, and biomass), photosynthetic characteristics (gas exchange parameters and photosynthetic pigments concentrations), antioxidant enzyme activities (SOD and CAT), ion homeostasis (K^+^ and Na^+^ transport, and K^+^/Na^+^ ratio), and osmotic adjustment (e.g. proline) in *G. inflata* were better than those in *G. uralensis*. These observations indicate that *G. inflata* is more salt tolerant than *G. uralensis*. Foliar application of Si effectively reduced the absorption of Na^+^, improved ion balance, alleviated membrane damage, and promoted the growth of the two liquorice species. Furthermore, the response of *G. uralensis* to Si was more pronounced than that of *G. inflata*, indicating that the protective effect of Si is different for different liquorice species. This study provides a theoretical basis for the evaluation of salt tolerance in and scientific artificial cultivation of liquorice in the future.

## Abbreviations

Si: Silicon
LEL: Leaf electrolyte leakage
ROS: Reactive oxygen species
SOD: Superoxide dismutase
CAT: Catalase
*P*_*n*_: Net photosynthesis rate
*T*_*r*_: Transpiration rate
*g*_*s*_: Stomatal conductance
*C*_*i*_: Intercellular CO_2_ concentration
Chl: Chlorophyll
MDA: Malondialdehyde
Rubisco: Ribulose bisphosphate carboxylase oxygenase
